# New opportunities for RGD-engineered metal nanoparticles in cancer

**DOI:** 10.1186/s12943-023-01784-0

**Published:** 2023-05-25

**Authors:** Wei Qin, Jyoti Chandra, Mohammed A.S. Abourehab, Neelima Gupta, Zhe-Sheng Chen, Prashant Kesharwani, Hui-Ling Cao

**Affiliations:** 1 Xi’an Key Laboratory of Basic and Translation of Cardiovascular Metabolic Disease, College of Pharmacy, Xi’an Medical University, Xi’an, 710021, China; 2 Department of Pharmaceutics, School of Pharmaceutical Education and Research, Jamia Hamdard, New Delhi, 110062 India; 3 Department of Pharmaceutics, College of Pharmacy, Umm Al-Qura University, Makkah, 21955 Saudi Arabia; 4 Dr. Harisingh Gour Vishwavidyalaya (A Central University), Sagar, Madhya Pradesh 470003 India; 5 Institute for Biotechnology, College of Pharmacy and Health Sciences, St. John’s University, Queens, New York 11439 USA; 6 Center for Transdisciplinary Research, Department of Pharmacology, Saveetha Dental College, Saveetha Institute of Medical and Technical science, Chennai, India

**Keywords:** Cancer imaging, cancer, Metal nanoparticles, RGD, Integrin, Drug targeting

## Abstract

The advent of nanotechnology has opened new possibilities for bioimaging. Metal nanoparticles (such as gold, silver, iron, copper, etc.) hold tremendous potential and offer enormous opportunities for imaging and diagnostics due to their broad optical characteristics, ease of manufacturing technique, and simple surface modification. The arginine-glycine-aspartate (RGD) peptide is a three-amino acid sequence that seems to have a considerably greater ability to adhere to integrin adhesion molecules that exclusively express on tumour cells. RGD peptides act as the efficient tailoring ligand with a variety of benefits including non-toxicity, greater precision, rapid clearance, etc. This review focuses on the possibility of non-invasive cancer imaging using metal nanoparticles with RGD assistance.

**Graphical abstract:**

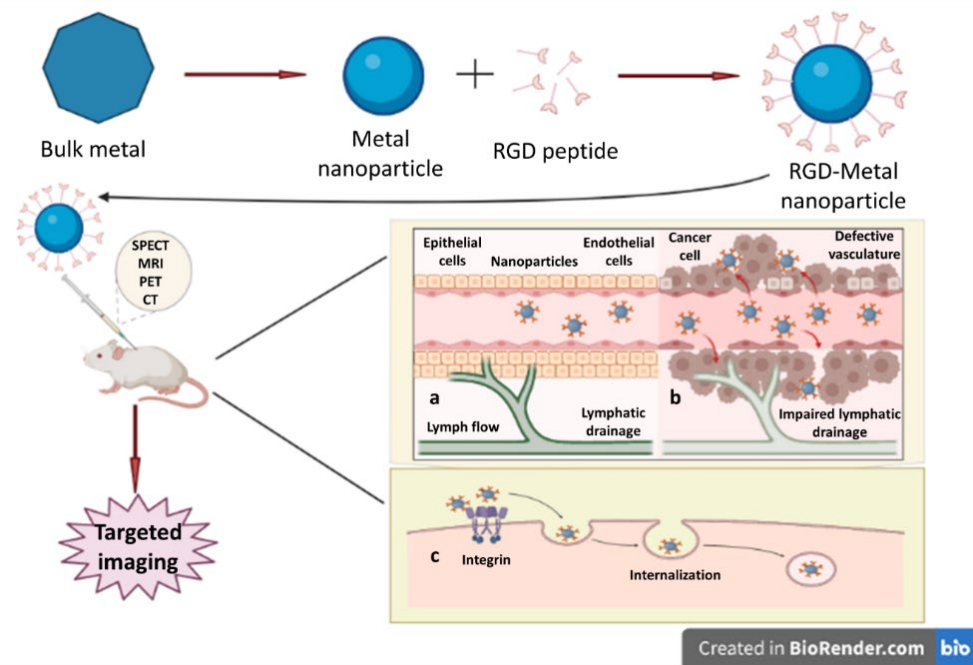

## Introduction

Biomedical imaging is the cornerstone of integrative patient care due to its ability to provide real-time surveillance, ease of use without damaging healthy tissue, low level of invasiveness, and applicability across several timeframe and size levels. Protein interaction and chemical interactions might take milliseconds, while illnesses like cancer can take decades to develop [1].

The use of biomedical imaging in treating cancer is expanding at every stage of treatment. Predicting, screening, and biopsy advice for the diagnosis, prognosis, treatment planning, therapy assistance, treatment outcomes, and recurrence are some of the few stages that require imaging [2–10]. Healthcare professionals can use a variety of imaging techniques, such as X-ray (plain film and computed tomography [CT]), ultrasound (US), magnetic resonance imaging (MRI), single-photon emission CT (SPECT), positron emission tomography (PET), and optical imaging while diagnosing, staging, and treating human cancers. Merely 4 modalities (CT, MRI, SPECT, and PET) have the potential to detect cancer in three-dimensions (3D) across the body. There have been tremendous advancements in diagnostic radiography, yet human cancer diagnosis and visualisation have lagged [11].

Finding and detecting as few tumour cells as possible is the prime purpose of cancer imaging, preferably even before the angiogenic switch is turned on [12–17]. The definitions of “imaging” and “detection” are rather arbitrary, depending on the voxel dimensions of the specific imaging technique in question. Though a cluster of cancerous cells on the subvoxel scale would be detected, it would not contribute to the formation of a 3D picture as it would occupy just a solitary voxel. No matter which measurement is employed, the detection threshold continues to be of the utmost significance. The existing prediction threshold for solid tumours is sadly around 10^9^ cells developing as a solid structure. One gramme is equal to one centimetre in volume. Therefore, from an imaging aspect, the phrase “remission” essentially indicates that there are between zero and 10^9^ malignant cells in the body of the patient who is undergoing treatment. This degree of unpredictability cannot be tolerated by either the patient or the caregiver [11]. This review aims to describe various arginine-glycine-aspartate (RGD)-based metal nanoparticles as prospective imaging agents in particular forms of cancer by targeting integrins effectively.

**Table 1:** RGD-based metal nanoparticles for cancer imaging

S. No	Type of nanoparticle	Targeting motif	Cancer	Purpose	Cell line (In vitro)	Animal model (In vivo)	Outcome of study	Ref
1	Au	RGD	Glioblastoma, melanoma	SPECT/CT imaging	M21, M21-L, U87MG	M21, M21-L, U87MG xenografts in athymic Nude-Foxn1nu mice	Improved targeting capability was achieved	[93]
2.	Au-PAMAM	cRGD	Solid tumors	SPECT/CT imaging	C6		Improved SPECT/CT imaging was achieved with good stability and biocompatibility	[100]
3	Ag_2_S	c(RGDfk)	Solid tumors	Fluorescence/electron microscopy	4T1luc, A549, e A431	4T1luc tumor-bearing BALB/c	Enhanced fluorescence enabled tracking single molecule in a cell without premature opsonization	[113]
4	Ag_2_S/BSA	RGD		Fluorescence/photoacoustic imaging & chemo/photothermal therapy	HeLa		Tailored chemo/photothermal treatment and fluorescence/photoacoustic imaging were effectively combined to provide a fully featured theranostic framework.	[114]
5	INP	RGD	Glioblastoma	MRI	U87MG	U87MG xenografts in BALB/c nude mice	Using multimodal approaches, tumour identification with a high degree of sensitivity was accomplished.	[124]
6	INP/DOTA	c(RGDyK)	Glioblastoma	PET/MRI	U87MG	U87MG xenografts in mice	Enhanced T1-weighted MR imaging was achieved	[126]
7	CuS	cRGD	Gastric cancer	PET imaging & Photothermal therapy	MKN45	MKN45 xenografts in female nude mice	Non-invasive monitoring and mapping of metastatic lymph nodes were achieved	[153]
8	ErNPs@MnO_2_	RGD	Triple Negative Breast Cancer	NIR-II imaging and gene delivery	MDA-MB-231	MDA-MB-231 xenografts in BALB/c nude mice	Therapy using sensitive NIR-II imaging with GSH response was accomplished	[159]
9	Pd/Chitosan	c(RGDfK)	Triple Negative Breast Cancer	NIR imaging	MDA-MB-231, HEK-293, MG-63	MDA-MB-231 xenografts in BALB/c nude mice	Utilizing a non-invasive near-infrared laser, improved tumour imaging and treatment were accomplished.	[180]
10	Tc/Ornithine	c(RGDfK)		SPECT/MRI	U87MG	U87MG xenografts in SCID mice	Significant tumour uptake led to improved SPECT/MR imaging	[190]

**List of abbreviations used in the table: Au –** Gold NPs, **Au-PAMAM –** Gold- poly(amidoamine) NPs, **Ag**_**2**_**S** – Silver sulphide NPs, **Ag**_**2**_**S/BSA –** Silver sulphide/Bovine serum albumin NPs, **INP** – Iron oxide NPs, **INP/DOTA** - Dodecane tetraacetic acid/Iron NPs, **CuS** – Copper sulphide NPs, **ErNPs@MnO**_**2**_ – Erbium loaded manganese oxide NPs, **Pd/Chitosan** – Palladium loaded chitosan NPs, **Tc/Ornithine** – Technetium/ornithine NPs, **RGD** - Arginyl-glycyl-aspartic acid, **cRGD –** Cyclic Arginyl-glycyl-aspartic acid, **c(RGDfk)** - Cyclo (Arg-Gly-Asp-D-Phe-Lys), **c(RGDyK) -** Cyclo(Arg-Gly-Asp- [D-Tyr]-Lys), **SPECT/CT** - Single-photon emission computerized tomography/Computed tomography, **MRI** – Magnetic resonance imaging, **PET/MRI** - Positron emission tomography/Magnetic resonance imaging, **NIR** – Near infrared

## Nanoparticle-based cancer imaging

Recent developments in molecular imaging and nanotechnology are opening up new possibilities for bioimaging and holding tremendous potential for the creation of drugs to meet clinical demands for disease staging, differentiation, and the tracking of treatment outcomes [18–26]. Nanomaterials have special optical, magnetic, and chemical characteristics that make it possible to design imaging probes with higher pulse densities, intensified signals and measurement, better contrast, and optimised biodistribution [27–36].

It is crucial to develop a nanoparticle imaging agent in a way that promotes its delivery to a malignant lesion for maximising the diagnostic performance of the agent [37–39]. Interestingly, the main benefit of targeted nanotechnology is its architectural freedom. Fabrication of nanoparticles for numerous clinical imaging technologies is made possible by an abundance of components, including lipids, metals, and polymers. The use of targeted nanoparticle imaging agents offers a fresh perspective on cancer imaging that goes beyond morphological characterisation and allows for both molecular/cellular assessment of therapeutic efficacy and early cancer identification [15, 29, 40–42]. Nanoparticles allow the attachment of thousands of imaging moieties per structure, which can increase the signals by as much as a factor of a million. From the standpoint of research and development, nanoparticulate agents used in cancer imaging can be split into two groups: those that target blood vessels and those that selectively target cancerous cells. In regards, a variety of nanoparticle-based targeted agents have been created for use with various detection methods [19–24, 29, 30, 37, 43–45] (Table 1).

## Metal-based cancer imaging

Researchers have been curious about metallic nanoparticles (NPs) for almost a decade, and now they find widespread application in fields as diverse as biomedicine and technology. Their immense promise in nanotechnology has made them an interesting area of research. Metal nanoparticles are versatile entities that present several opportunities in a wide range of biological applications. These opportunities include diagnostic assays, augmentation of radiation, and thermal ablation [46–50]. This is due to the presence of a powerful electromagnetic field on the particle surface of the metal NPs, as well as their broad optical characteristics, ease of manufacturing technique, straightforward surface chemistry, and easy surface modification [51–53]. When compared to traditional therapeutic interventions, nanomaterials offer theranostics, which is a simultaneous screening and therapy technique that is directed at real-time surveillance of cancer treatment. This allows for a therapy that is more effective while carrying a lower risk than is possible with traditional therapies. The excellent tunable optical characteristics of metallic NPs, such as those made of gold, silver, or a blend of the two, allow them to be conveniently adjusted to the needed wavelength range depending on shape, size, and composition, making them appropriate for image processing and photothermal uses in local tissue [54, 55]. Noble metallic NPs are also capable of thermally destroying cancerous cells by effectively converting illumination or radiofrequency energy into heat [56, 57]. Moreover, metallic NPs with greater density are easier to be absorbed by cells compared with non-metallic NPs of comparable sizes, which is an advantageous technique for cancer therapy [58].

In addition, various imaging methods have been developed over the period of several decades to visualise various clinical conditions. These imaging methods diverge in both their instrumentation and methods, and more importantly, they require a contrast agent with specific physicochemical characteristics. As a consequence of this, various NP-based contrast agents, such as metallic NPs, were identified and developed for use in the aforementioned imaging technologies [59]. There is a possibility that imaging is either an indirect or direct action of NPs. In the event of direct action, NPs can be used in conjunction with specialist equipment such as heat analyzers, MRI scanners, and fluorescence microscopes to locate tumour cells. Some nanoparticles, such as gold, silver, and nanoparticles made of supramagnetic iron, can emit luminescence or chromogen in living systems [60]. In the instance of indirect activity, the NPs contribute to the bioimaging of tumour cells, yet they do not exhibit luminescence or chromogenic activity by themselves within the biological system; rather, they contain reacting chromogens, enzymes, antibodies, substrates, or reactive chromogen. Because of this, the chromogen-loaded NPs interact with the precise biological system that these NPs enter [60].

Surface modification of metal nanoparticles with specific targeting ligands enables the imaging of biological processes in a more precise manner and thereby improves the effectiveness of the imaging method.

## RGD-based cancer imaging

**Fig. 1:**
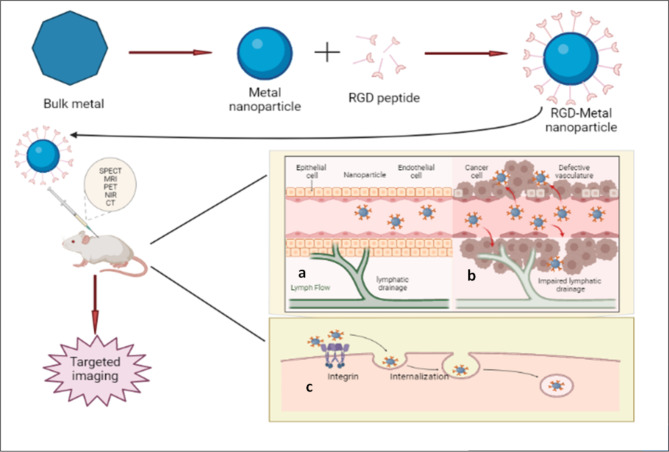
Graphical representation of RGD modified metal nanoparticles for cancer imaging. Figure representing receptor mediated endocytosis of RGD conjugated metal nanoparticle for targeted imaging with different modalities. (**a**) Normal cell, (**b**) Tumour cell with leaky vasculature, (**c**) Receptor mediated endocytosis

Angiogenesis, or the creation of fresh blood vessels from vasculature that is already present, is an important step in various biological processes, including female reproductive functionality, development, and tissue repair. In addition, disruption of the normal angiogenesis pathways plays a part in the development of numerous disorders, including cancer, psoriasis, arthritis, and retinopathies, which manifest as excessive growth of blood vessels. Integrins are a crucial part of the process of angiogenesis. Numerous peptide receptors, including integrins, have been discovered to be highly expressed in cancerous cells [35, 61, 62]. The integrin family controls several vital biological processes during the onset, development, and spread of tumours [63–65]. αvβ3 is a key integrin implicated in tumour growth, neovascularisation, and migration, and the expression level of this molecule is correlated with cancer progression [66–68]. About two decades ago, the RGD motif was identified as a cellular attachment ligand that predominantly binds to αvβ3 integrin receptors [69, 70]. In tumour cells, integrin αvβ3 expresses exclusively, while it is barely detectable in healthy cells. The upregulation of αvβ3 during tumorigenesis, infiltration, and spreading was therefore proposed as a predictive biomarker for cancer, and it represented an appealing in vivo molecular target for earlier cancer detection and targeted therapy [71, 72] (Fig. 1). For example, RGD-modified silica nanoparticles reported by Li et al. demonstrated excellent targeted ability in visualising prostate cancer cells with higher fluorescence intensity in vivo [73].

**Fig. 2:**
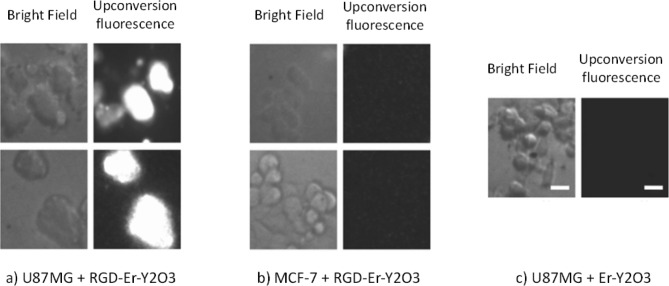
Fluorescence images of in vitro staining of human glioblastoma U87MG (high integrin αvβ3 expression) and human breast cancer MCF-7 (integrin αvβ3 negative) using RGD-modified UCP nanoparticles. Reproduced with permission from ref [77]

The use of peptides for targeted delivery offers a variety of excellent benefits, such as metabolic consistency, rigidity to structural modification, non-toxicity, greater precision, rapid clearance, no immunogenic response, simple fabrication, and countless conjugation options to imaging and therapeutic agents [74, 75]. RGD peptides both in their linear and cyclic variants are now often utilised for imaging and therapy. Moreover, when compared to the linear version of RGD, cyclic RGD seems to have a considerably greater ability to adhere to integrin adhesion molecules. Therefore, to deliver the payload to the area of αvβ3-overexpressing tumour cells, RGD motifs thus act as an efficient tailoring ligand [76]. The same can be inferred from work by Zako et al. who reported the creation of cyclic RGD-modified upconversion (UC) nanophosphors composed of erbium-yttrium oxide (Er-Y_2_O_3_) nanoparticles for visualising tumour cells [77]. Rare earth ion-containing ceramics are known as UC phosphors. By sequentially activating different rare earth ion energy levels, the compounds may collect infrared radiation and transform it to produce visible light [78]. When integrin-negative MCF-7 cells and integrin-positive U87MG cells were used to compare the UC emission of RGD-Er-Y_2_O_3_ with IR excitation, it became clear that MCF-7 did not exhibit any UC fluorescence from RGD-Er-Y_2_O_3_ NPs. U87MG cells, however, were easily discernible. Moreover, RGD-integrin interaction was further confirmed in the case of U87MG cells that did not show any fluorescence in the case of non-targeted Er-Y_2_O_3_ NPs (Fig. 2) [77]. Due to the wide range of applicability for infrared-to-visible fluorescence, cellular imaging with UC phosphors is of high relevance. Visualising integrin αvβ3 expression employing UC phosphors probes will have significant promise for cancer imaging. The following sections discuss an overview of several RGD-modified metal nanoparticles, as well as their potential in the delivery of a variety of imaging entities.

### RGD-modified gold nanoparticles

Over countless generations, gold has served as a cornerstone of science and technology. While a relatively inert metal, the characteristics of gold are entirely altered at the nanoscale range because of the radical modifications in its electrical performance at that scale [9, 79–83]. Among the various nanoparticles, colloidal gold nanoparticles (AuNPs) are considered to be robust nanoparticles that have a variety of intriguing characteristics. In Egypt and China, AuNPs may have been used for ornamentation and medical care in as early as the fourth or fifth century B.C. [84].

The incorporation of AuNPs in biomedical research has attracted attention in recent years due to their inherent characteristics that render them suited for cancer therapy and detection. Surface plasmon resonance (SPR) is responsible for giving AuNPs their one-of-a-kind characteristics. Because the interactions are seen among the electromagnetic field of light and the conducting ions of AuNPs, electrons immediately start oscillating in resonant frequencies when illumination is imposed on AuNPs at a particular wavelength. As a result of this property, they can be employed in a wide variety of biomedical imaging techniques, including MRI, photoacoustic imaging (PAI), fluorescence, and X-ray scatter imaging [85–87].

**Fig. 3:**
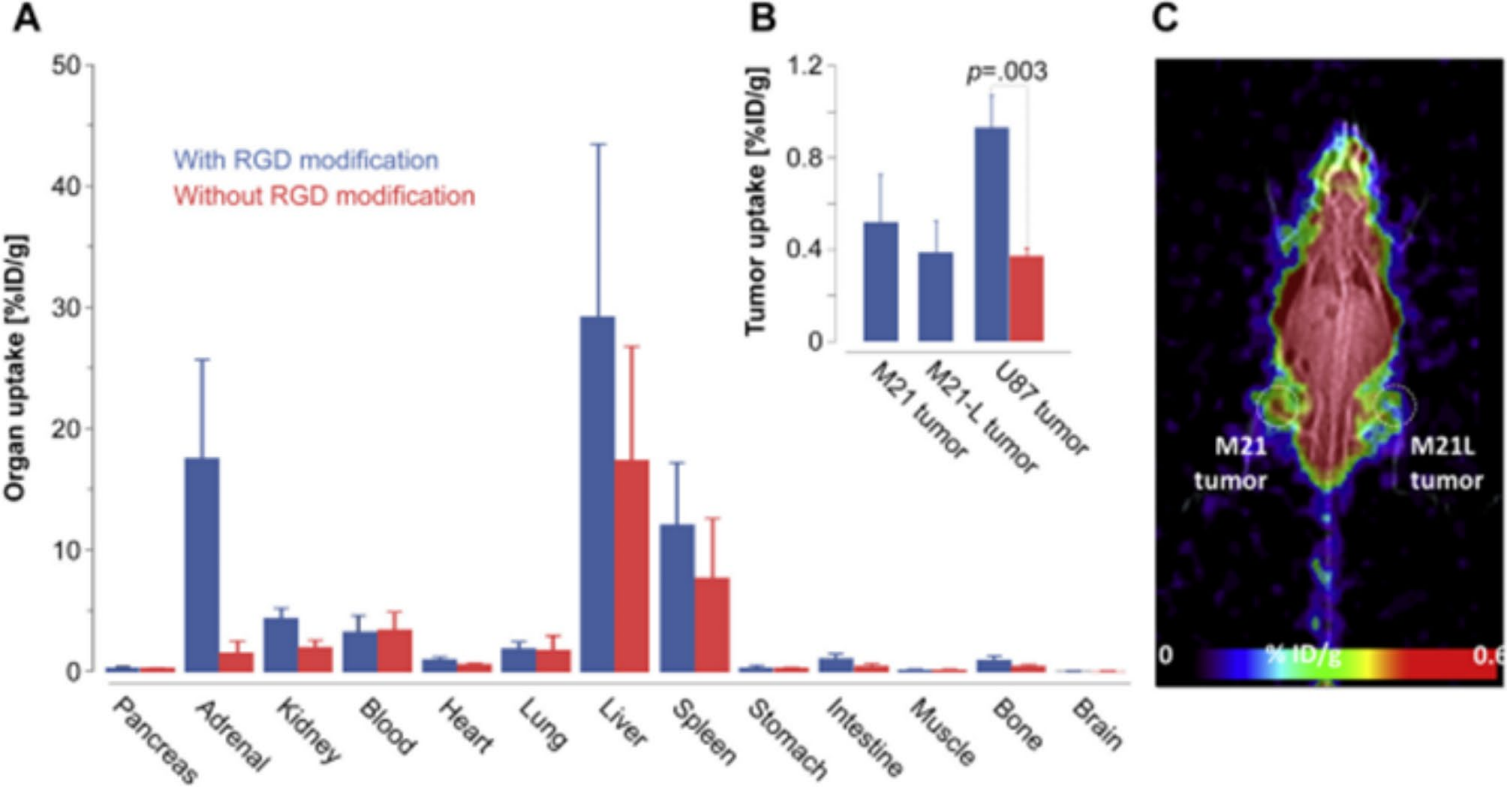
In vivo targeting with RGD-modified indium-111 labeled gold nanoparticles. Biodistribution studies were performed in M21, M21-L and U87 tumor xenografts. The organ (**A**) and tumor biodistribution (**B**) demonstrated higher tumor uptake with RGD modified indium-111 labeled gold nanoparticles when compared with the non-targeted nanoparticles in the U87 tumor model. SPECT/CT imaging (**C**) demonstrated higher uptake of RGD-modified indium-111 labeled gold nanoparticles in the M21 tumor (left) compared to the M21-L tumor (right). Reproduced with permission from ref [93]

The increasing need for imaging that is selective to biological mechanisms has prompted the clinical application of a wide variety of radiotracers. Long-term imaging tracers must be optimised for several parameters to be effective, including greater labelling yield, increased labelling stability, and minimal intrusion with biological characteristics [88]. Methods of radiolabeling with a mono-radioactive atom include conjugation and chelation with metallic chelating agents [89–91]. However, in practice, the fraction of conjugated and complexed atoms in a molecule is typically less than one. In addition, chelation using radioisotopes can indeed be difficult, necessitating extreme reaction mechanisms, which could be disruptive to the biological characteristics of the tagged compounds [92]. As a direct consequence of this, the timeframe for diagnostic practice using radioisotopes is typically no longer than a couple of hours. Imaging techniques that enable the labelling of high activities while retaining strong labelling stability and causing minimal interaction with the biological features of the target are widely pursued to expand these periods [93]. Avoiding metal chelating agents and severe radiolabelling methods by incorporating radioisotopes directly onto AuNPs frees up the whole particle surface for alterations using targeting ligands and thus, maximises the efficiency of the process. Quinn et al. have described the construction of a molecular visualising framework which was based on AuNPs explicitly labelled with indium-111 that have had their surfaces changed with RGD motifs for molecular localisation within αvβ3 adhesion molecules. Upon evaluating tumour accumulation and biodistribution of indium-111–tagged AuNP with and without RGD alteration in female athymic nude Foxn1nu mice harbouring human melanoma M21, M21-L, or U87-MG glioblastoma xenografts. Absorption of cRGD-modified particles was found to be greater in M21 tumours that exhibited elevated levels of αvβ3 integrin than in M21-L tumours that expressed low levels of αvβ3 integrin, suggesting that αvβ3 integrin is responsible for tumour adsorption. Moreover, in agreement with the biodistribution findings, SPECT/CT visualisation showed that the M21 tumour absorbed more RGD-conjugated indium-111–tagged AuNPs than the M21-L tumour (Fig. 3) [93].

In most cases, contrast molecules are required for superior cellular imaging. Multifunctional molecular probes made from a wide range of nanomaterials have been developed, manufactured, and deployed for synergistic scanning [94–97]. The choice of a flexible, easily-functionalized nanoplatform is vital for the development of a multifaceted NP system [98]. Dendrimers made from poly(amidoamine) (PAMAM) can be used as a template to create multimodal contrast agents because of their cavity structure and high concentration of amine groups at the periphery [99]. In fact, generation 5 (G5) PAMAM dendrimers altered with chelators and targeting ligands can be utilised as templates for the manufacture of AuNPs with surface-modification using Gd(III) ions via chelation for CT/MR scanning of malignancies. To facilitate tumour SPECT/CT imaging, Xu et al. produced G2 dendrimers encapsulated with AuNPs tagged with technetium [100]. The NPs were then attached through a PEG linkage to RGD and 2,2′-(7-(2-(2,5-dioxopyrrolidin-1-yl) oxy) -1,4,7 triazonane-1,4-diyl)diacetic acid (NOTA-NHS ester). As prepared, (Au)-G2-NOTA(99mTc)@RGD NPs were tested for cytocompatibility, and the results showed that C6 glioma cells treated with the NPs had a survival rate of greater than 94% at Au concentrations varying from 200 to 4000 nM. Additionally, it was also demonstrated (Au)-G2-NOTA(99mTc)@RGD NPs had promising SPECT imaging properties for integrin-expressing C6 cells when tested in vitro. Increasing the radiation dose yields a progressively brighter SPECT image. At a given radioactive dose, SPECT pictures of cells given targeted (Au)-G2-NOTA(99mTc)@RGD NPs were noticeably brighter than those of cells treated with non-functionalised counterparts. Moreover, findings from SPECT imaging in vivo were consistent with in vitro studies, confirming the potential of RGD conjugation as a viable approach for directed SPECT/CT imaging of malignancies that express αvβ3 integrin [100].

In another study, Chen et al. reported the development of RGD peptide-modified versatile dendrimer-encapsulated AuNPs loaded with Gd for selective MRI and CT [101]. RGD-dependent targeting selectivity of the Gd/Au/DNPs@RGD probe to αvβ3 integrin-expressing cancerous cells was confirmed by their absorption in vitro. When compared to standard L1210 cells lacking αvβ3 integrin activity, U87MG cells accumulated Au at a rate that was 2.2% and 3.9% greater at 10- and 40-mM Au, respectively. At 10- and 40-mM Au concentrations, U87MG cells preincubated using plain RGD showed significantly reduced Au absorption than the ordinary U87MG cells, indicating that unbound RGD treatment inhibits surface αvβ3 integrin expression, which reduces Au absorption. In addition, CT imaging performed in vivo showed a significant CT contrast improvement in the tumour site at 1, 3, and 20 h after injection compared to preinjection scanning. The CT value of the tumour area decreased 20 h after administration, indicating that the NPs can be digested over a period of time. The selectivity of RGD-dependent localisation was validated by preinjection of plain RGD, which was thought to inhibit αvβ3 receptor activity in the tumour area. At various times after administration, tumour regions in mice given plain RGD showed significantly lower CT contrast augmentation than tumour regions in animals not treated with plain RGD. Consistent with CT imaging findings, MR imaging also revealed an uptick in MR signal amplitude in the case of RGD-functionalised NPs compared with their non-targeted counterparts, thus presenting a potential strategy of dual-modality tailored CT/MR scanning of various cancers with overexpression of the αvβ3 integrin [101].

### RGD-modified silver nanoparticles

Silver NPs are tiny silver particles, ranging in size from 1 to 100 nm. Certain materials, despite being widely referred to as “silver,“ include a significant amount of silver oxide because of the enormous proportion of surface-to-bulk silver atoms. Ionic silver seems to have a lengthy history and was first employed in yellow-stain glasses, similar to AuNPs [102–104].

With a broad array of detecting modalities, including colourimetric, scattering, SERS, and MEF techniques, silver NPs have various advantageous optical features at incredibly lower detection levels that have led to the development of novel strategies in sensing and imaging applications. Additionally, the utilisation of scattering imaging methods has been achieved by NPs which has facilitated the improvement in conventional imaging methods. Single silver NPs are an excellent choice for molecular labelling because of their SPR and enormous efficient scattering cross-Sects.  [105, 106]. Thus, the development of numerous tailored silver oxide nanoprobes is underway.

**Fig. 4:**
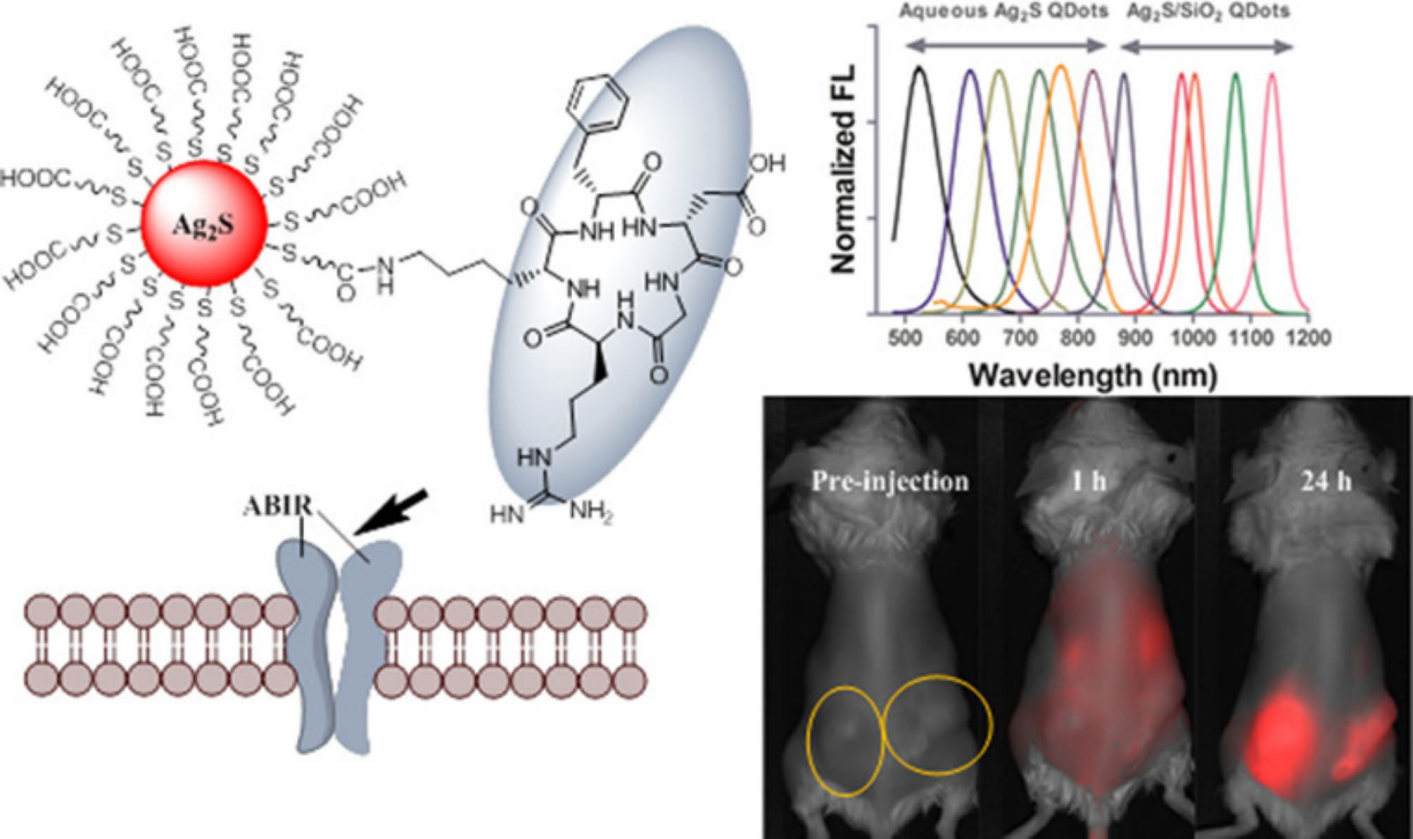
Schematic representation for the formation of silver sulfide quantum dots for integrin-targeted cancer imaging. Reproduced with permission from ref [113]

Quantum dots (QDs) and other fluorescent nanoparticles are frequently utilised in biomedical imaging due to their ability to circumvent problems inherent in more traditional organic fluorescent materials [107–109]. Even while cadmium- and indium-based QDs have the aforesaid useful characteristics, there are still some reservations about employing them in biomedical imaging. The polymer coating has been effectively used to reduce possible toxicities and offers functionalized anchoring for these QDs [110, 111]. Unfortunately, this method not just considerably increases hydrodynamic size (> 20 nm), but also makes the production process more difficult and sometimes uses chemicals that could be hazardous to the body. Giant QDs have the additional drawback of being largely limited to the vascular compartment, which limits their ability to extravasate from blood arteries and diffuse to cells located outside of the vasculature. Because of their huge size and protracted stay in the systemic circulation, they are more apt to be opsonised and to be taken up more readily by the liver’s reticuloendothelial system (RES). In imaging investigations, such changes may result in cytotoxicity or decrease the effectiveness and sensitivity of QD [112]. Consequently, using an orthogonal strategy, Tang et al. created RGD-attached water-soluble silver-sulphide QDs with a broad spectrum emission (520–1150 nm) for targeted transport into tumour cells [113]. Results from fluorescence and electron imaging revealed that only 4T1luc breast cancer cells incubated with the RGD-tagged QDs exhibited preferential integrin-mediated uptake. However, in up to 24 h of exposure, time-lapse imaging demonstrated that only a small percentage of non-targeted QDs were being taken up by cells. This is analogous to the response of tiny, negatively charged hydrophilic dyes, which are repelled by the charge-charge interaction between themselves and the negatively charged phosphate-rich cytoplasmic membrane and hence do not enter the cells. I.V. administration of QDs in various mouse cancer models demonstrated a large tumour-to-liver absorption ratio, indicating that the tiny diameter of the QDs prevented them from being opsonised and subsequently being taken up in large quantities by the liver and spleen. The application of the RGD-based nano-architectures for cellular imaging of illnesses and the tracking of therapy effectiveness is supported by the quick and selective absorption of such QDs in malignancies [113] (Fig. 4).

On a similar line, Yin et al. constructed RGD-conjugated theranostic nanoarchitectures with doxorubicin (Ag_2_Se@BSA/DX QDs) for integrated FL/PA imaging and tailored chemo/photothermal treatment [114]. Confocal laser microscopy was utilised to qualitatively analyze Ag_2_Se@BSA/DX QDs with HeLa cells using the typical red fluorescence of DX. This allowed them to determine whether or not Ag2Se@BSA/DX QDs could be employed for in vitro fluorescence imaging. In comparison to plain DX and Ag_2_Se@BSA/DX QDs, the red fluorescence intensity from Ag_2_Se@BSA/DX QDs was the brightest, indicating preferential absorption by HeLa cells due to the presence of RGD peptide. Furthermore, when cilengitide, an antagonist for integrin, was introduced to cells before therapy, DX fluorescent signal reduced dramatically compared with the group lacking cilengitide, implying that the blocker prevented RGD receptor-mediated endocytosis. This finding showed that RGD functionalization on QDs could increase phagocytosis. Moreover, in most instances, photothermal conversion molecules also have the capacity for PAI. The group further investigated the PAI properties of Ag_2_Se@BSA/DX QD after being prompted by the aforementioned concept. Investigational results showed that the photoacoustic patterns brighten and the related photoacoustic signal amplitude intensifies as the Ag^+^ concentration rises, postulating that Ag_2_Se@BSA/DX QDs can perform admirably as a PAI agent for cancer treatment when the process is directed by PAI [114].

### RGD-modified iron oxide nanoparticles

Iron is a fundamental component of all living things; hence, it is impossible to sustain life in its absence. Iron oxide nanoparticles, also known as IONPs, have a multitude of possible uses in nanomedicine, including cancer treatment, drug transport, and biomedical imaging. Due to this, they are driving a lot of optimism in the medical field [115–117]. The centre of IONPs is generally a magnetic iron oxide (magnetite, Fe_3_O_4_, or maghemite, Fe_2_O_3_), and they are stabilised by a protective layer that can be formed using a wide range of chemical methods [118, 119].

MRI represents one of the most robust minimally invasive visualising modalities because of its outstanding spatial magnification, tomographic characteristics, and capacity to gather 3D tomographic data with significant soft tissue differentiation and contrast [120]. Nevertheless, to achieve superior levels of sensitivity along with accuracy in MRI modalities, it is indeed necessary to increase the distinction between healthy and unhealthy tissues. Therefore, the utilisation of external MR contrast agents is truly unavoidable. The majority of the MR contrasting compounds that are presently available are either paramagnetic gadolinium or manganese chelate-based preparations. However, the freshly documented cases of nephrogenic system fibrosis that were related to Gd(III)-based contrast agents generated major safety issues, particularly in individuals whose renal function was aberrant [121–123]. Therefore, to get around the issues that are related to the contrasting agents currently available, Luo et al. created RGD-conjugated super tiny INPs for directed T1-weighted positive MR imaging of glioblastoma that overexpresses αvβ3 integrins [124]. Results from the cytocompatibility study revealed that after being exposed either to conjugated or non-conjugated NPs, the vitality of U87MG cells was found to be reduced marginally on increasing Fe levels comparable to the PBS control. The viability tests on U87MG cells showed that 80.0% were still alive after being injected with INP-mPEG or INP-PEG@RGD NPs at the maximum Fe levels investigated, suggesting acceptable cytocompatibility of INP-mPEG or INP-PEG@RGD. Researchers were able to find that while using INP-PEG@RGD NPs as a nanoprobe for directed T1-w MR imaging of tumours, the MR distinction of U87MG tumours was significantly improved. The highest buildup of the NPs inside the tumour location occurred at 45 min postinjection, allowing for the greatest MR contrast augmentation at the tumour site which could be attributed to RGD-mediated endocytotic uptake in integrin-overexpressing tumour locations [124].

In terms of anatomical, physiological, and resolution aspects, each molecular diagnostic technique provides an exclusive array of benefits and limitations. Multilabelled imaging probes have allowed for the simultaneous assessment of a single target molecule by two or more detection methods. This enables the synergistic use of each technique’s capabilities, which ultimately results in improved diagnostic performance and a deeper understanding of pathogenic mechanisms [125]. Even though multimodal visualising techniques are employed in modern medical settings to obtain a more exact multiparametric picture of illnesses, distinct imaging materials are normally employed for individual modalities. As a result, it would be beneficial to construct one probe which could perform several functions and could also be detectable by multiple imaging modalities simultaneously. In light of recent developments in nanotechnology, ligand-based NPs might prove to be the most suitable foundation upon which to build multimodal imaging molecules [25, 125]. Thus, Lee et al. created an RGD-conjugated INP-based MRI/PET dual functionality probe for tumour visualisation of integrin αvβ3 expression, making use of the great sensitivity of PET and the superior spatial resolution of MRI [126]. Synthesis of INPs was accomplished by using a polyaspartic acid (PASP) covering and the macrocyclic chelating agent dodecane tetraacetic acid (DOTA). It was determined through the use of αvβ3-positive U87MG cells in a displacement competitive binding test that DOTA/INP@RGD can bind exclusively to integrin αvβ3 adhesion molecules in vitro. In addition, the monomeric c(RGDyK) and DOTA/INP served as controls for the experiment. The DOTA/INP@RGDs were successful in inhibiting the attachment of ^125^I-echhistatin to the integrins αvβ3 that were expressed on U87MG cells. Furthermore, integrin-specific transport of functionalised RGD@PASP/INP nanoparticles was demonstrated by both small-animal PET and T2-w MRI. In addition to shedding light on the molecular processes of malignancy, the effectiveness of such dual-functionality imaging technology might even permit earlier, more accurate tumour diagnosis [126].

Among the most prevalent types of cancer, nasopharyngeal carcinoma (NPC) is notable for its high degree of vascularisation. Management of advanced NPC has been shown to be successful through an emphasis on angiogenic determinants, using antiangiogenic medicines either alone or in combination with chemo or radiation therapy [127, 128]. The use of a particular antiangiogenic medication in such a therapy, unfortunately, might not be advantageous for all individuals [129]. Accordingly, noninvasive visualisation of tumour angiogenesis is not only a sophisticated method for evaluating the initial clinical efficiency but also for tailoring antiangiogenic therapies by optimising dosing levels and duration of the medication cycle [130, 131]. To aid in the timely detection of tumour angiogenesis, numerous RGD-based platforms have been created for targeting integrins in particular [132]. MRI is a noninvasive imaging modality that can locate both superficial and deep cancers without causing any damage to the patient. Its benefits include superior spatial resolution and strong soft tissue contrast. Yet, MRI’s limited usefulness for bioimaging is due to the technology’s intrinsic insensitivity. Because of this, negative MR compounds with significant relativities based on the inherent characteristics and variable surface functioning of ultrasmall superparamagnetic iron oxide (USPIO) nanoparticles are an exciting new area of research [133, 134]. To detect tumour angiogenesis in human NPC, Cui et al. produced a new polyacrylic acid (PA)-coated USPIO NPswhich were conjugated with RGD (USPIO@RGD/PA) [135]. The selective binding of USPIO@RGD/PA for integrin αvβ3 was confirmed by in vitro cell-line investigation, which showed that integrin-expressing HUVECs exhibited greater absorption of USPIO@RGD/PA and could be lowered following treatment with unbound RGDs. In addition, an in vivo test was carried out using CNE-2 tumour models wherein integrin αvβ3 was exclusively localised on the vascular wall of the tumour. The findings of the experiment showed that USPIO@RGD/PA induced a diverse signal drop primarily at the periphery and a few central portions of the tumour. This is compatible with the patterns of profuse angiogenesis and αvβ3 integrin expression of the cancerous cells. On contrary, mice that merely had non-targeted USPIO showed only a moderate decrease in T2 signal, which can most likely be attributed to the extravasation of nanoparticles from intra-tumoural arteries and their subsequent distribution across the interstitium. Quantitative analysis indicated that the T2 relaxation period was reduced substantially postinjection of USPIO@RGD/PA than postinjection of simple particles. The result was further validated by Prussian blue staining of tumour tissues that demonstrated that the majority of USPIO@RGD/PA NPs travelled and accumulated in tumour angiogenic arteries, whereas pure USPIO extravasated into the extravascular space. Altogether, these results suggested that USPIO@RGD/PA can rapidly and accurately assess tumour angiogenesis in a human NPC xenograft model utilising MRI [135].

**Fig. 5:**
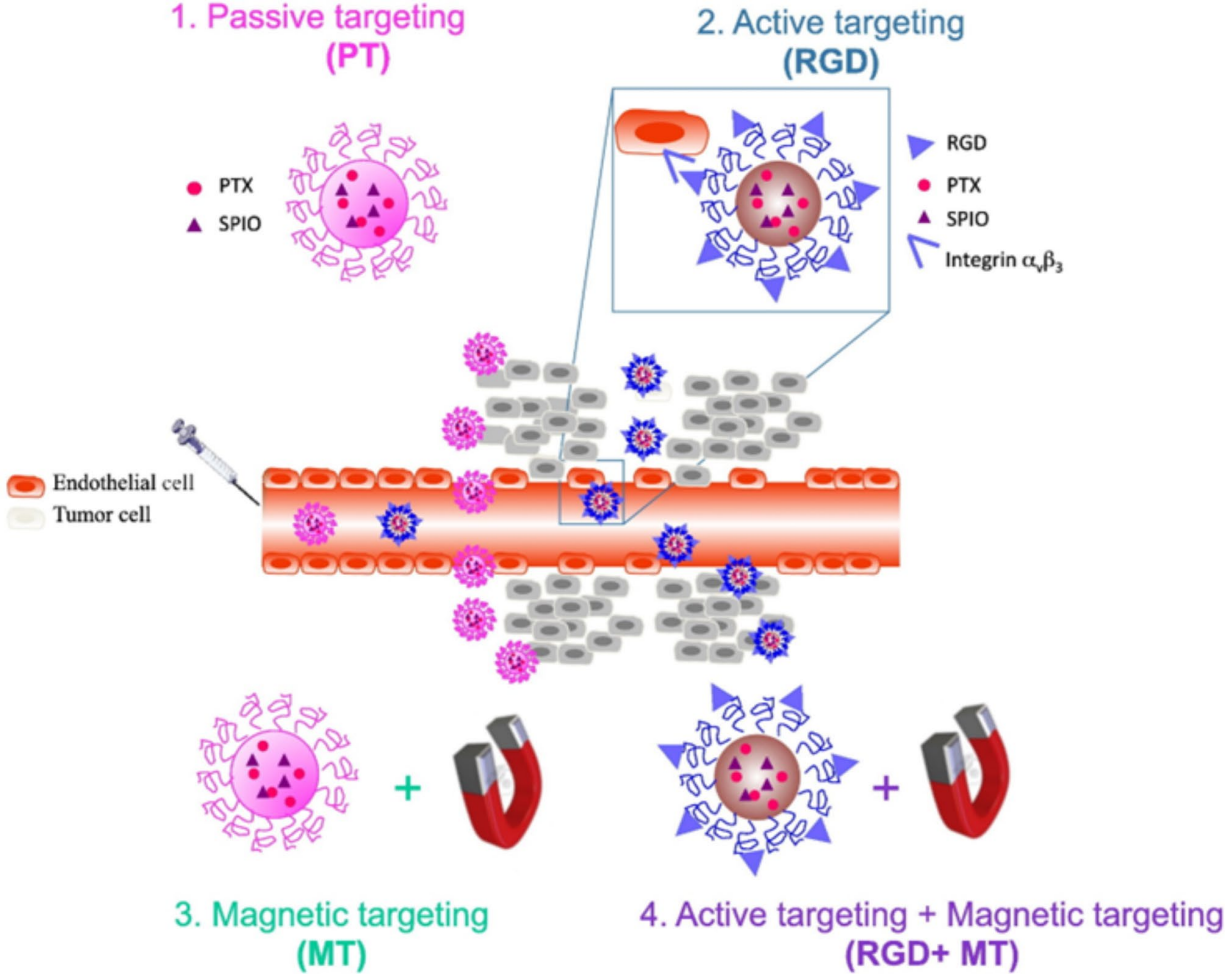
Schematic representation of the different tumor targeting strategies compared in the present study. (1) Passive targeting via the EPR effect (PT), (2) active targeting of αvβ3 integrin via RGD grafting (RGD), (3) magnetic targeting via a magnet of 1.1 T placed on the tumor (MT) and (4) the combination of magnetic targeting and active targeting of αvβ3 integrin (RGD + MT). Reproduced with permission from ref [136]

In another piece of research, Schleich et al. constructed RGD-based therapeutic and diagnostic platforms utilising SPIO nanoparticles that were loaded with paclitaxel for the purpose of scanning and treating tumours (SPIO/PX@RGD) [136]. In vivo MRI was performed on mice before and 4 h after the administration of NPs. The results demonstrated that non-targeted SPIO/PX NPs did not generate any apparent darkening of the tumour site. While mice injected with SPIO/PX@RGD showed a small darkening of the tumour area along with several darker patches placed into the malignant cells, non-targeted NPs exhibited no effect on the tumour. This influence was indicated by a propensity that increased RSD by a factor of three postinjection. Besides, mice who received treatment with SPIO/PX and SPIO/PX@RGD, which were steered by an outside magnetic field created by putting a 1.1 T neodyme iron bore magnet, demonstrated a significant difference in RSD. In the instance of SPIO/PX, a substantial number of dark patches could be seen across the tumour site, which resulted in a thirty-fold jump in RSD postinjection. Combining magnetic targeting and active targeting generated a significant darkened contrast, which resulted in a forty-fold improvement in RSD postinjection in the case of SPIO/PX@RGD [136] (Fig. 5).

### RGD-modified copper nanoparticles

In recent years, there has been a rise in research into the potential medical benefits of copper nanoparticles [137]. Copper-based chalcogenide semiconducting NPs, and more specifically copper monosulfide (CuS) NPs, have evolved into a major category of photothermal conducting agents in competition with AuNPs. Considering the translational ability of CuS NPs versus AuNPs, the former offers several significant benefits [138–143]. As a preliminary step, their absorbance can be fine-tuned by controlling particle size, although they are very mildly impacted by post-production treatments and their natural environment [144]. Secondly, unlike other gold nanoarchitectures, near-infrared (NIR)-absorbing CuS NPs can be formed into a significantly smaller size (20 nm), which might also result in more favorable pharmacokinetic and elimination qualities and increased tumour cell localisation [138]. Finally, radioactive [^64^Cu]CuS-NP can be developed for PET scanning and quantitative cell evaluation because they can incorporate positron-emitting ^64^Cu radionuclide during fabrication by avoiding the incorporation of radioactive chelating agents [138]. In addition to CuS NPs, various copper chalcogenide nanostructures have been studied for possible application in medical diagnostics and photothermal therapy (PTT), including copper selenide nanocrystals, copper telluride nanostructures, and copper oxide NPs, among others. In addition, cancerous cells can be imaged with the help of copper nanoclusters’ fluorescent capabilities [145, 146].

**Fig. 6:**
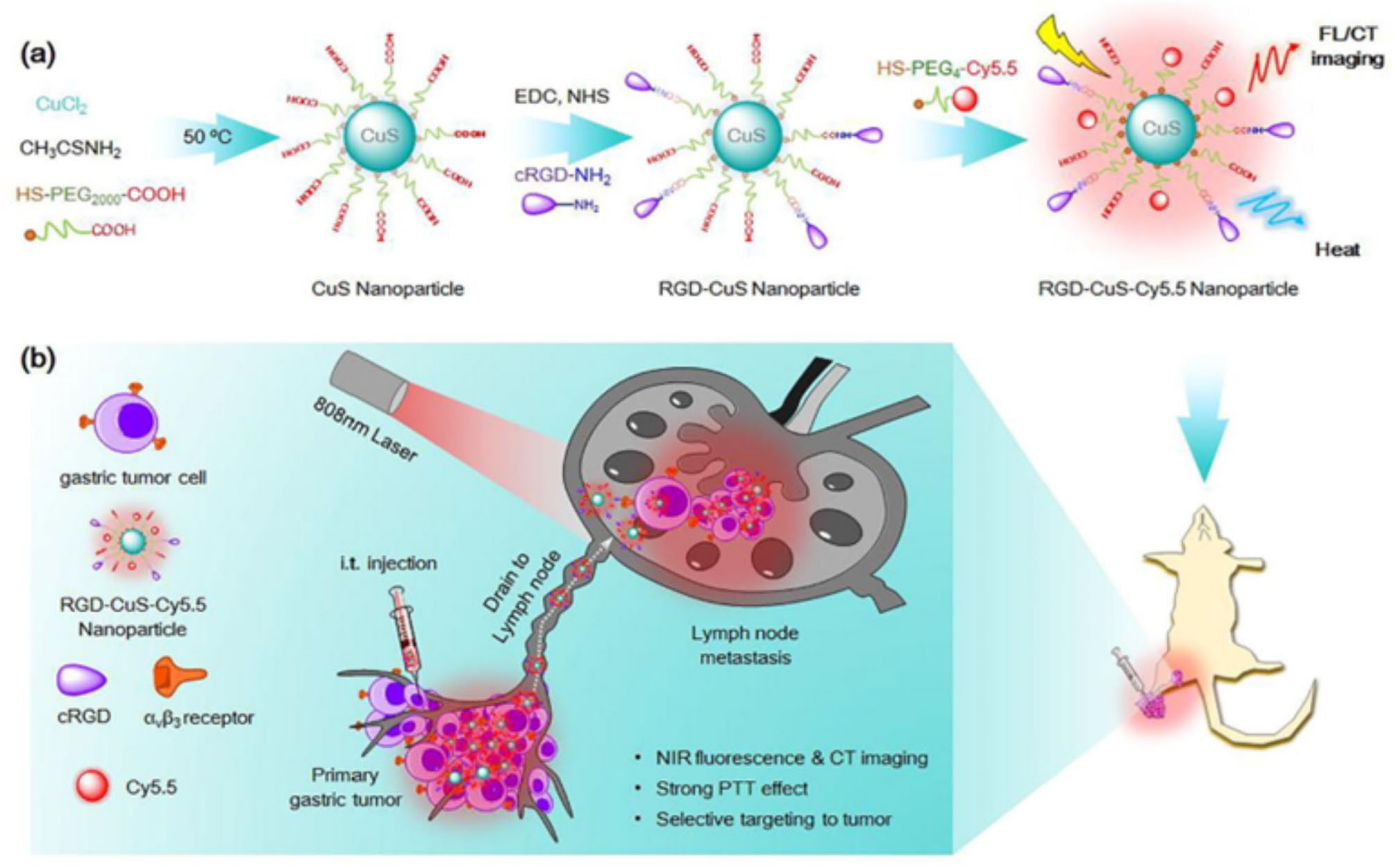
General design of fluorescent RGD-CuS-Cy5.5 nanoparticles. (**a**) Schematic illustration of the design and synthesis for RGD-CuS-Cy5.5 nanoparticles consisting of strong NIR absorbing CuS nanoparticles, sensitive NIR fluorophore Cy5.5 and a tumor targeting ligand cRGD. (**b**) Illustration of the utility of RGD-CuS-Cy5.5 for imaging-guided photothermal therapy of lymph node metastasis of gastric tumor in vivo. After i.t. injection of RGD-CuS-Cy5.5, they easily drain to lymph node due to the small nanoparticle size. The following recognition by the integrin αvβ3 overexpressed in gastric tumor cells can trigger efficient cell uptake via receptor-mediated endocytosis and subsequently accumulate in the lymph node, resulting in strong NIR fluorescence and CT contrast indicative of lymph node metastasis. Irradiation of the lymph node with an 808 nm laser to perform PTT process can cause significantly elevated temperature, which in turn triggers irreversible tumor cell death. Reproduced with permission from ref [153]

Among the most frequent types of cancer, gastric cancer is the third greatest cause of death from cancer around the globe [147]. It has been discovered that the likelihood of stomach cancer spreading to the lymph nodes is very significant [148]. To direct the subsequent treatment and prevent the spread of cancerous cells, it is crucial to distinguish between malignant and non-malignant lymph nodes in individuals. Currently, the sole treatment option for stomach cancer in health centres is the surgical removal of both primary cancer and malignant lymph nodes; regrettably, this procedure carries postsurgical hazards and complications, such as lymphorrhoea, pancreatic fistula, and gastric abscess, which could also intensify inflammatory response, bump up the chance of death, and lower the likelihood of long-term survival [149–152]. Therefore, there is a great need for novel treatment approaches that allow for the precise excision of malignant gastric cancerous cells from the lymph nodes while causing the least amount of damage to healthy tissue. Therefore, as an alternative to surgical dissection, Shi et al. created fluorescent CuS nanoparticles by incorporating a tumour-targeting cRGD and NIR organic dye, Cy5.5 [153]. They used these nanoparticles for paired fluorescence/CT bimodal imaging and preferential photothermal treatment of gastric tumour progression in sentinel lymph nodes (SLN). Researchers had revealed that RGD@CuS/C5.5 had significant NIR absorbency, intense fluorescence, and X-ray attenuation, providing an exceptional photothermal characteristic for efficient PTT and dual-modal fluorescent/CT signals for in vivo imaging of SLN invasion. Additionally, the preferential absorption into αvβ3-overexpressing cancer cells could be triggered by the covalent attachment of cRGD motifs to the surface of RGD@CuS/C5.5. RGD@CuS/C5.5 was effectively able to penetrate the SLN and was directed to the proliferative MKN45 tumour cells in living animals, as evident from in vivo investigations. This led to high luminescence and CT visual signals that may effectively guide PTT of malignant tumours. A new effective strategy for the surgery of gastric malignant transformation has been made possible by the successful development of fluorescent RGD@CuS/C5.5 NP that enable both SLN metastasis tracking and targeted PTT in vivo (Fig. 6) [153].

### RGD-modified lanthanide nanoparticles

Lanthanides are a group of chemical elements that have been distinguished by atomic numbers varying between 57 (lanthanum) to 71 (lutetium). They are often regarded as “rare earth” metals since it was originally believed that there was only a tiny quantity of them available within the outer layer of the Earth’s crust [154]. Even though lanthanides in their pure state are highly prone to instability, they are found frequently in rocks, ores, and minerals in variety of oxides and fluorides. Since the procedures for the recovery and purification of lanthanides minerals keep on improving, an increasing number of researchers are concentrating their efforts on the potential applications of such metals in the diagnosis and treatment of cancer. The oxidation/reduction stability of Ln^3+^ ions renders them well suited to be used in cellular domains in the presence of physiological reductants such as ascorbate and thiols. Additionally, these ions have desirable luminescent features that can be attributed to electron transfer, 4f-5d, and f-f shifts [155]. Radioisotopes derived from lanthanides, such as ^177^Lu, are presently being employed for diagnostic and therapeutic purposes in the field of oncology. Nanoparticles, nanodrums, and nanocrystals of lanthanide oxide have all shown their potential as diagnostic entities and prospective chemotherapeutic medicines [156, 157]. Nanoparticles made of Ln_2_O_3_ are of particular relevance in molecular diagnosis because of the exceptional imaging capabilities of lanthanides which result from their considerable paramagnetic potential at ambient temperature [158]. The improvement of biocompatibility is indeed the primary concern for nanomedical studies conducted before clinical trials. The body’s natural response to everything it perceives to be alien is rejection, so biomaterials are no exception. To be effective in vivo, a molecule must be designed to be non-toxic, to remain in the bloodstream for an extended period of time, and to be either biodegradable or sufficiently tiny to be easily removed by the kidneys. RGD-based surface functionalization of lanthanide nanomaterials to make them more biocompatible is a rapidly growing area of study in biomedical engineering [158].

For pinpoint tumour localization, lanthanide fluorescence nanoprobes have been extensively produced and explored due to their broad-spectrum reconfigurable regions and extremely sensitive imaging. Because of the extensive penetration depth, NIR fluorescent scanning is commonly employed in this frequency region for tumour identification. Luminescence in the second NIR window (NIR-II) allows for higher-resolution scanning since it penetrates deeper into cells and has less autofluorescence than probes generated in the first NIR range. The development of a nanoprobe that can be triggered just at the tumour location by coupling with MnO_2_ would be a significant step forward in accurate tumour localization. Therefore, nanoarchitectures (ErNP@MnO_2_/S100A4-RGD) based on RGD-conjugation on manganese dioxide (MnO_2_) lanthanide nanoprobes of erbium (Er) for tumour imaging and delivering S100A4 siRNA in triple-negative breast cancer (TNBC) were developed by Ming et al. [159]. ErNP@MnO_2_-RGD were shown to accurately image tumours upon administration into MDA-MB-231 xenograft-carrying nude mice at a volume of 200 µL, as confirmed by in vivo findings. ErNP@MnO_2_ altered with RGD motifs were more concentrated in the tumour area than the unaltered counterparts, and mice injected with ErNP@MnO_2_ produced dim fluorescence at the tumour location. Their investigation suggested that high-resolution NIR-II bioimaging of tumours might be accomplished using ErNP@MnO_2_-RGD [159].

Glioblastoma multiforme (GBM), the most frequent and lethal major brain tumour in humans, is responsible for between 15% and 20% of all intracranial tumours and 50% of all cerebral gliomas [160]. As per the World Health Organization’s (WHO) categorisation of tumours of the central nervous system, GBM is categorised as a category IV glioma despite its extreme aggressiveness, high recurrence, and poor prognosis [161, 162]. This is because of its dismal prognosis. Surgical tumour excision is the initial step in treating glioblastomas since it can alleviate symptoms by reducing the size of the tumour tissues, improving the accuracy of the pathological diagnosis, improving the efficacy of subsequent chemoradiotherapy, and increasing the survival rate [163]. Entire tumour excision is seldom performed, nevertheless, because of the infiltrative characteristics of the tumour and the possibility of harming the surrounding functioning brain cells. To visualise the tumour lesion in GBMs before surgical removal, a non-invasive MRI technique is generally employed [164]. Along with MRI scans before the surgery, fluorescent-guided excision has lately been designed to assist surgeons in accomplishing aggressive GBM removal [165]. The red fluorescent protoporphyrin IX (PpIX), which glows red when illuminated with blue light, accumulates in the GBM regions using the chemical 5-aminolevulinic acid (ALA), according to this method. Blue excitation, meanwhile, presents significant drawbacks, including intense autofluorescence and limited cellular infiltration, when contrasted against NIR illumination. The photo-bleaching and low photo-stability of PpIX are additional problems. NPs, lanthanide emission, and UC luminescence features are all integrated into lanthanide-doped UC nanoparticles (UCNPs) [166]. To convert NIR photons into the visible range, absorption occurs successively. Background luminescence is insignificant when excited at 980 nm because the majority of biomolecules have little absorption and scattering in the NIR range (700–1000 nm). Additionally, this illumination can penetrate deeper tissue, minimising phototoxicity to living things. Therefore, in single modality UC fluorescence scanning of cells, UCNPs have been coupled to a variety of biocompatible compounds [167]. Thus, to better visualise xenografts of human brain tumours, Jin et al. developed RGD-modified UC nanoparticles coupled with Gd^3+^-DOTA [168]. MRI was used to examine the in vivo targeting properties of UNP/Gd@RGD in U87MG tumour xenografts at a gadolinium dose of 0.0125 mmol kg^− 1^. According to the study’s findings, UNP/Gd did not produce any visible MR signal amplification in tumours, and the tumours’ margins remained unclear throughout the scanning time. The main explanation is that the UNP/Gd group utilised a very small amount of Gd^3+^-DOTA, as little as 1/8 of the recommended dose. The tumour zone of the UNP/Gd@RGD group, in comparison, consistently displayed an evident MR increase despite a minimal Gd^3+^-DOTA dose, and the borderline between the tumour and nearby healthy cells was well demarcated with great contrast. Further evidence that UNP/Gd@RGD targets glioblastoma is provided by the researcher’s observation of a strong augmentation at 60 min p.i. compared with at 30 min p.i., and the MR increment fading steadily after a 24-hour injection. This targeting potential is the result of the combination of integrin receptor recognition and the EPR effect. In addition, *ex-vivo* UC imaging showed that the UC fluorescence intensity in the U87MG tumour of the UNP/Gd@RGD group was indeed considerably greater than that in the UNP/Gd group, indicating its immense promise in directed MRI of glioblastoma and in UC fluorescent signal-guided excision [168].

In a different work, Cao et al. created lanthanide nanoparticles (UNP@P/RGD/NGR) coated in polydopamine that were combined with RGD and NGR for dual targeting-based cancer imaging [169]. Comparing UNP@P/RGD/NGR to UNP@PDA, quantitative analysis of cellular accumulation in A549 cells revealed that uptake was increasing, with an 11-fold rise in Y^3+^ absorption at the dosage of 160 m Y^3+^. Findings of an in vivo investigation on tumour-targeted UC luminescence (UCL) in BALB/c nude mice showed that at 15 and 30 min following UCNP@PDA injection, a diffuse purple fluorescence was seen. In contrast, in the instance of UNP@P/RGD/NGR, substantially stronger blue-violet UCL via 980 nm excitation from the tumour location of the treated mice was detected. Since UNP@P/RGD/NGR slowly spread out from the tumour location to certain other locations, the amplitude of the UCL at a later period was smaller compared to the earlier period. The margin of the tumour was clearly distinguishable from the region of the tumour that was generating fluorescence, therefore confirming the higher selectivity of UNP@P/RGD/NGR for A549 cells. Additionally, UNP@P/RGD/NGR displayed no significant toxicity when compared to the control group, demonstrating tremendous promise for real-time intraoperative mapping [169].

### RGD-modified palladium nanoparticles

NPs derived from noble metals have found increasing application in fields as diverse as illness detection, medication transport, cancer cell therapy, and biomedical imaging, with the introduction of improved mass-produced submicron technologies. Prospective biomedical utilisation of NPs has piqued the interest of scientists due to their diameter, optical and electronic characteristics, and molecular shapes [170]. Nanomaterials founded on palladium (Pd) have demonstrated promising biological applications over the last few years owing to extremely inexpensive cost, excellent heat resistance, strong chemical stability, outstanding photocatalytic characteristics, electrical features, and optical functionalities [171, 172]. Even though Pd-based nanomaterials were developed much later than other extensively researched noble nanomaterials like Au and Ag, their unique properties—including their strong photoconversion effectiveness and excellent photothermal stability—have garnered significant interest in the area of nanotechnology [173]. In light of these distinguishing characteristics, Pd-based nanoparticles have emerged as a promising candidate for use as contrast agents in tumour visualisation and therapy for the illness.

**Fig. 7:**
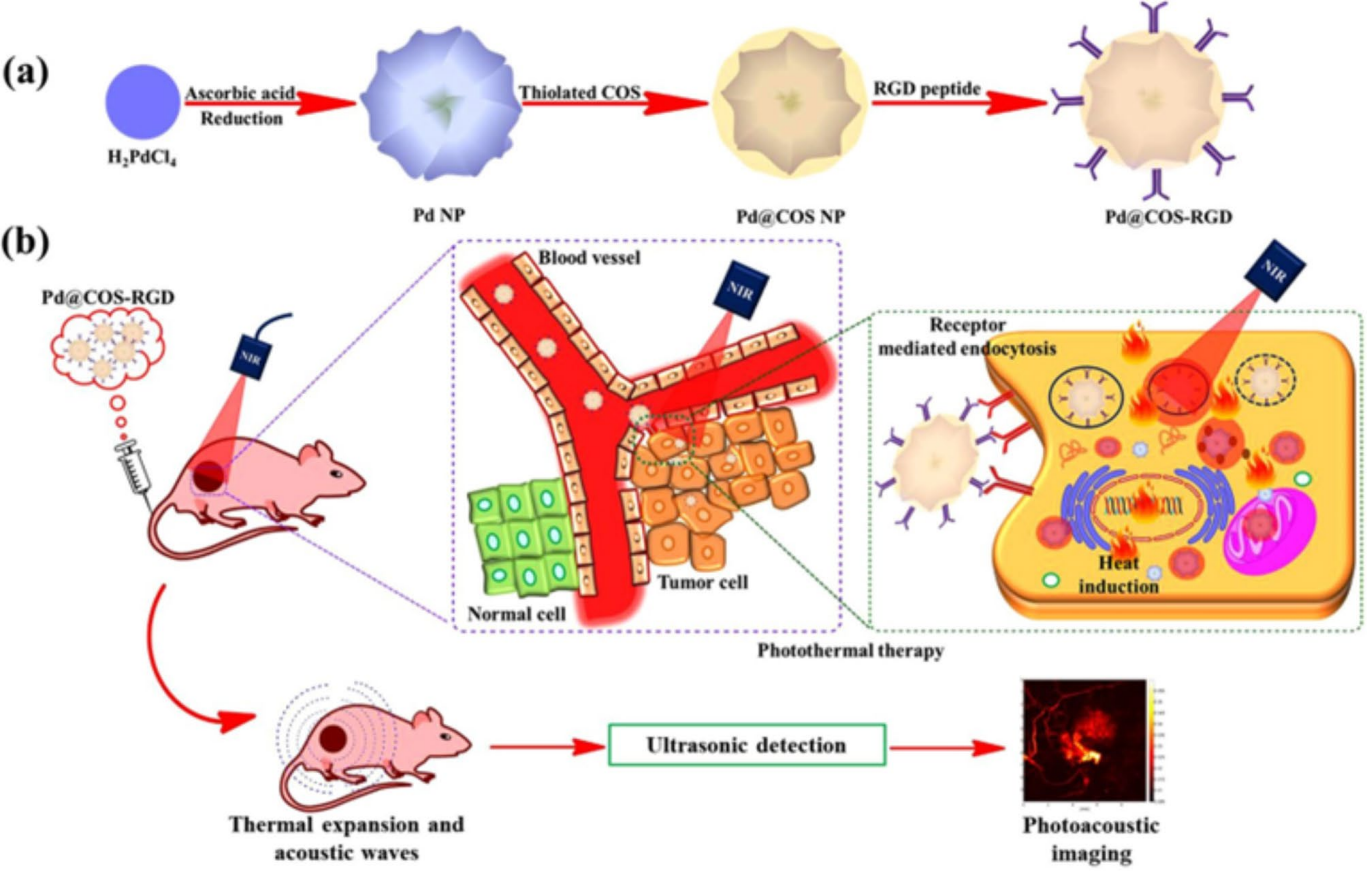
(**a**) A scheme showing the preparation of Pd NPs and further surface coating with thiloated chitosan oligosaccharide (Pd@COS NPs) and finally functionalization using RGD peptide (Pd@COS-RGD). (**b**) A systematic illustration showing the photothermal ablation and photoacoustic imaging of tumor tissue using Pd@COS-RGD. Reproduced with permission from ref [180]

PTT, a recently established technique that uses NIR laser photo-absorbers to produce heat under NIR laser illumination, has drawn great attention in Pd nanoparticles (Pd NPs) exhibiting NIR absorption. PTT seems to have several benefits versus traditional chemotherapy, namely excellent specificity, less intrusiveness, accurate spatial-temporal precision, and efficient removal of malignant cells [174–176]. PTT can also be used in tandem alongside multimodal imaging to track progress and even actualise a treatment goal. The ability of photoacoustic tomography (PAT) to provide non-invasive monitoring free of ionising rays and cellular damage has increasingly caught the attention of researchers in biomedicine. PAT allows for high-resolution, both spatially and temporally, visualisation of deep tissues. In PAT setups, nanomaterial contrast agents can generate a strong response at the target region [177, 178]. Very little research has been done on PAT effectiveness of Pd NPs. Pd NPs can be used for PAT because of their high NIR absorption spectrum, which can be paired with imaging for medical reasons. Because of the short-wavelength attenuation of cells in the NIR spectral range, high-resolution scanning of tissues with extrinsic contrast media can be achieved [179]. Surface modification is indeed an essential stage in the process of controlling the interactions that nanoparticles have with the human body. Purposeful fabrications like RGD-based surface modifications provide the tailored motifs for targeted attachment of nanostructures on the cells, which in turn increases the nanoparticles’ potential to be internalised by specific cells. For instance, Bharathiraja et al. have developed multimodal RGD-based Pd NPs with chitosan oligosaccharide for photoimaging and treatment [180]. This team investigated the PAT effectiveness of Pd/ChOS@RGD in vitro and in vivo due to the positive photothermal properties of Pd. During in vitro PAT investigation, a phantom was designed to simulate living cells. After 4 h of treatment using Pd/ChOS@RGD (10 and 50 ppm), MDA-MB-231 cells were introduced into the simulator with 8% gelatin and compared to the non-treated control sample. An increased PA pulse intensity was seen in Pd/ChOS@RGD-labelled cells, allowing them to be differentiated from the dummy background. Since cells do not absorb NIR light, a rise in NP level was associated with a corresponding increment rise in the PA signal intensity, which was not observed in the untreated group. Moreover, as an added measure, PAT was conducted in vivo both before and following injection of Pd/ChOS@RGD into mouse tail veins for a period of one hour. The results showed that Pd/ChOS@RGD localised in the tumour area and produced increased PA impulses after being administered. Pd/ChOS@RGD’s potential as an effective contrast medium is demonstrated by the higher intensity PA signals observed in treated mice, which were not present in control mice. The RGD-functionalised nanoprobe exhibited tumour-targeting potential, which aided in the simple detection of cancerous cells [180] (Fig. 7).

### RGD-modified technetium nanoparticles

Technetium-99 m, also known as ^99m^Tc, is a radionuclide that emits and has a broad array of clinical use in molecular imaging. It is utilised in both planar scintigraphy and single PET because it possesses the optimum nuclear characteristics for efficient visualisation with gamma cameras [181]. Systemically delivered ^99m^Tc radiopharmaceuticals are designed to concentrate within the targeted location. The level of radioactivity that has localised at the targeted site is proportional to how well the organ functions, which enables the diagnosis of a particular condition [181]. The radionuclide’s nuclear characteristics, the rate at which it accumulates inside the targeted site, as well as the extent to which it is removed from the milieu significantly influence the visual resolution. When compared to other detection methods, radionuclide scanning is unmatched in its capability to identify metabolic/functional changes at the submicromolar or subnanomolar levels. Radiopharmaceutical formulations typically employ a ^99m^Tc dosage of around 108 M. Unlike many other PET isotopes, which decay quickly, this one has a sufficiently long half-life to permit scanning over later periods as well [182].

**Fig. 8:**
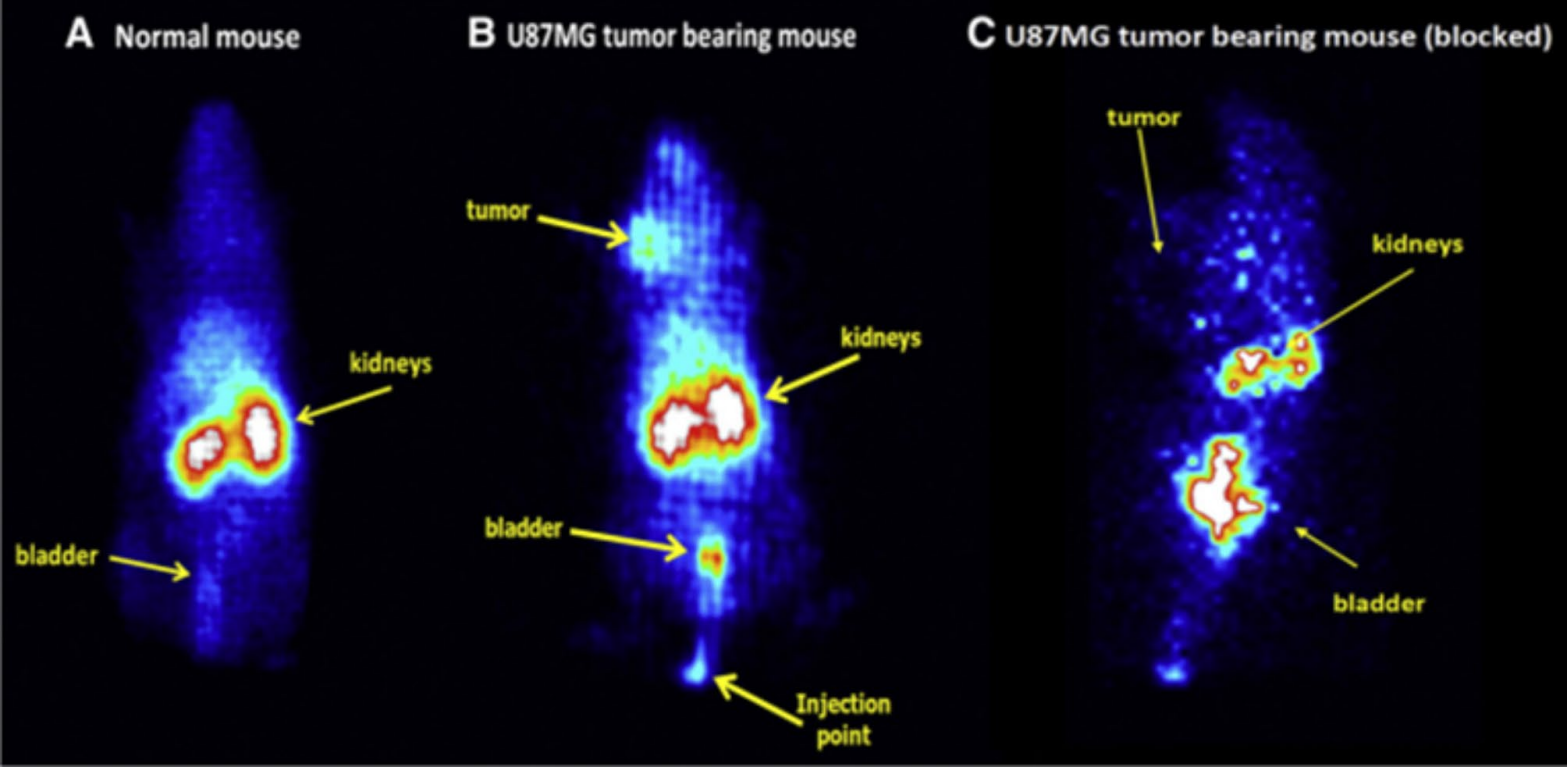
Planar γ images of c(RGDfK)@(Or)3-CGG/^99m^Tc): (**a**) a normal mouse, (**b**) a U87MG tumor-bearing mouse (**c**) a blocked tumor bearing mouse (U87MG onto the left front flank) after co-administration of an excess amount of the non-radiolabeled RGD derivative at 60 min p.i. The arrows indicate the presence of the tumor (or blocked tumor), kidneys, bladder and injection point. Reproduced with permission from ref [190]

It has been documented that radiopharmaceuticals based on the RGD motif exhibit great affinity and specificity for the αvβ3 adhesion molecules, making them suitable for the noninvasive screening of tumour angiogenesis. To create angiogenesis-specific radiocompounds for detection (^125^I, ^99m^Tc, ^111^In, ^18^ F, and ^64^Cu) and tumour-targeting radiation treatment (^90^Y and ^177^Lu), many RGD motifs and non-peptide RGD synthetic derivatives have been labelled with various radionuclides over the previous few years [182–189]. Radiolabelled cyclic RGDs have a significant problem when it comes to radiotherapeutics because of the substantial renal absorption and protracted retention of radioisotopes. Therefore, Tsiapa et al. created a novel hydrophilic RGD analogue with ornithine and arginine, called c(RGDfK)@(Or)3-CGG, that was further tagged by ^99m^Tc, in an attempt to optimise renal clearance and minimise upper abdomen region radioactivity [190]. In healthy and U87MG tumour-carrying SCID mice, biodistribution and dynamic-camera imaging experiments demonstrated immediate and substantial tumour absorption, along with rapid blood clearance and renal excretion. Furthermore, in a typical planar camera, scans of c(RGDfK)@(Or)3-CGG/^99m^Tc in normal mice and tumour-carrying models of U87MG were compared. The radioactivity accumulation was found to be similar in normal mice and the control tumour model, but the U87MG tumour could be distinguished with strong contrast, whereas the other SCID animal that had been given a large dose of untagged RGD combined with the radiolabelled RGDs for receptor blockage did not show any contrast in the same tumour (Fig. 8) [190].

Again, in a separate study, Tsiapa et al. reported the production of ^99m^Tc-labelled aminosilane-coated INP for use in bioimaging with c(RGDfK)@(Or)3-CGG [191]. It was validated from in vivo biodistribution experiments that the plain ^99m^Tc-NPs were weakly absorbed in the tumour, whereas RGD-conjugated ^99m^Tc-NPs presented 9-fold greater tumour uptake at 1 h postinjection. This was found to be the case when contrasting the two types of NPs. The deposition of either of the INPs in any of the other organs was virtually nonexistent. Investigations that involved blocking revealed a target selectivity for the integrin receptors found in U87MG glioblastoma cells. The initial in vivo research of an applied external magnetic field demonstrated that the induction of hyperthermia is achievable with the assistance of INPs. According to the data presented in the study, the RGD motifs that are attached to the NPs aim to bind to the αvβ3 integrins that are present in the malignant cells. It is anticipated that this particular accumulation, in conjunction with hyperthermia, will create an efficient platform for the treatment of malignancies and the identification of the disease [191].

## Conclusion

Many medical professionals and researchers have recently become interested in bioimaging, which enables non-invasive, repeated, and quantitative image analysis of specific targeting of biomolecules and viewing of diverse cellular mechanisms in progress. With the aid of cellular imaging, professionals can immediately comprehend the pathophysiology of an illness and determine the implicated pathways. Healthcare professionals use a variety of imaging modalities, and all of these methods are useful in understanding the physiological state of the body and differentiating between normal and unhealthy cells. The use of biomedical imaging in treating cancer is expanding at every stage of treatment, including predicting, screening, and biopsy advice for the diagnosis, prognosis, treatment planning, therapy assistance, and treatment outcomes. Finding and detecting as few tumour cells as possible is a prime purpose of cancer imaging, preferably even before the angiogenic switch is turned on. Nanoparticle-based imaging or more specifically, metal nanoparticle-based imaging, has become a versatile approach in the field of molecular imaging owing to its excellent optical features, powerful electromagnetic field, simple surface chemistry, and modification capabilities, thus offering real-time surveillance of cancer treatment as compared to traditional imaging techniques. However, it is crucial to develop a nanoparticle imaging agent in a way that promotes its delivery to a malignant lesion for maximising the diagnostic performance of the agent. Cancerous cells engaged in angiogenesis can be visualised using a targeting-based strategy and compounds designed specifically for imaging. For tumour cells to develop and proliferate, angiogenesis is an essential step. Integrins are extensively expressed on malignant cells, which, upon receiving signals, become triggered and facilitate the process, resulting in the stimulation of tumour-related growth factor. RGD peptides have been employed to target integrin receptors for many years. Various RGD-based metal nanoparticles have been discussed in this article and have shown success in targeting integrin overexpressed in cancer, with excellent imaging potential both in vitro as well as in vivo.

## Data Availability

Not applicable.

## Abbreviations

RGD: arginine-glycine-aspartate
CT: computed tomography
US: ultrasound
MRI: magnetic resonance imaging
SPECT: single-photon emission computed tomography
PET: positron emission tomography
